# Maternal perceptions and barriers experienced during the management of moderately malnourished children in northern Uganda

**DOI:** 10.1111/mcn.13022

**Published:** 2020-07-06

**Authors:** Richard Bazibu Kajjura, Frederick Johannes Veldman, Susanna Maria Kassier

**Affiliations:** ^1^ School of Health Science, College of Health Sciences Makerere University Kampala Uganda; ^2^ School of Health Care Sciences Sefako Makgatho Health Sciences University Pretoria South Africa; ^3^ School of Agricultural, Earth and Environmental Sciences, College of Agriculture, Engineering and Science University of KwaZulu‐Natal Pietermaritzburg South Africa

**Keywords:** malted sorghum, children, Uganda, maternal barriers, maternal perceptions, moderate acute malnutrition, supplementary porridges

## Abstract

Little is known about how the use of supplementary foods in the management of children with moderate acute malnutrition (MAM) is perceived by mothers, as well as the barriers they face while using it in Uganda. This study determined maternal perceptions and barriers experienced during the management of children aged 6 to 23 months diagnosed with MAM using either a malted sorghum‐based porridge (MSBP) or fortified corn soy blend (CSB+) as a supplementary porridge. Twelve focus groups and 48 in‐depth interviews were conducted among purposively sampled mothers a week after completing a 3‐month cluster randomised control trial with the two supplementary porridges for the management of their children with MAM in a rural setting of Arua district. All mothers who participated in the trial were eligible for inclusion. Mothers perceived both supplementary porridges as contributing towards weight gain, reducing the prevalence of illness, improving appetite, a healthy skin and improving active play. Barriers to using the supplementary porridges were a lack of time for feeding children due to household chores, maternal hunger as a result of household food insecurity and a lack of social household and community support. Therefore, maternal barriers should be addressed in an attempt to reap maximum benefits from supplementary food interventions for the management of children with MAM, by sensitising household members to the time required to conduct household chores and measure to address food insecurity.

**Key messages**

- Health workers should prioritise the nutrition education of all mothers and caregivers with children regarding appropriate complementary feeding practices delivered at health facilities and communal gathering places.
- Provision of supplementary foods for the management of malnourished children should be accompanied by sensitising mothers regarding the potential benefits for their children to counteract maternal barriers that could affect intervention implementation and impact optimal intervention outcome.
- There is need to document maternal perceptions and barriers experienced while using supplementary foods in the management of children diagnosed with MAM on a national level in urban, as well as rural settings.

## 1 INTRODUCTION

Globally, an estimated 35 million children under the age of five suffer from moderate acute malnutrition (MAM), with more than a quarter (27%) of all wasted children living in Africa (UNICEF, 2016). It is suggested that children younger than 2 years with MAM residing in Africa, seldom achieve their full physical, intellectual, or cognitive developmental potential (Cichon et al., 2016; Rytter, Kolte, Briend, Friis, & Christensen, 2014). More than two thirds of childhood deaths in low‐income countries are due to inappropriate complementary feeding practices (Lenters, Wazny, Webb, Ahmed, & Bhutta, 2013). In addition, in a low‐income country like Uganda, only 14% of those aged 6 to 23 months consume the minimum acceptable diet, thus contributing towards child mortality (UBOS & ICF, 2018).

Systematic reviews indicated that supplementary foods are increasingly used in the management of children aged 6 to 23 months with MAM (Annan, Webb, & Brown, 2014; Lazzerini, Rubert, & Pani, 2013). Moreover, the use of supplementary foods for managing MAM in low‐ and middle‐income counties (Lazzerini et al., 2013) has not adequately focused on maternal perceptions and barriers faced as a means of optimising the efficacy of supplementary foods (Ickes et al., 2012; Lazzerini et al., 2013), as it is dependant on caregiver use of these foods (Ickes et al., 2012). An Ethiopian study investigating caregivers' perception regarding the use of supplementary food to manage malnourished children documented that it was often used to meet household needs as a result of household food insecurity (Tadesse, Berhane, Hjern, Olsson, & Ekström, 2015).

Under certain circumstances, including poor management of MAM, children did not recover and regressed to severe acute malnutrition, whereas others defaulted, and some passed away (Lazzerini et al., 2013). The most commonly reported barriers to appropriate feeding practices among well‐nourished children, included a lack of time, household food insecurity and a lack of social support from household members (Ickes, Hurst, & Flax, 2015; Jones, Agudo, Galway, Bentley, & Pinstrup‐Andersen, 2012;Nankumbi & Muliira, 2015 ; Nor et al., 2012). It is evident that many children aged 6 to 23 months are not receiving adequate complementary food (Lazzerini et al., 2013), compounded by social and care issues as a result of socio‐economic status (Nankumbi & Muliira, 2015; Nor et al., 2012). In addition, some mothers may seek complementary treatment options such as traditional and spiritual healers as an adjunct to mainstream treatment options such as supplementary foods (Nor et al., 2012). It is also evident that the maternal decision to feed their children the supplementary foods they are issued with is based on whether they believe in the ability of these foods to treat MAM (Ickes, Wu, Mandel, & Roberts, 2018). However, little is known about maternal perceptions regarding the effectivity of the dietary management of their children diagnosed with MAM when using a supplementary porridge (Lenters et al., 2013). The findings of the current study provide insight into maternal perceptions regarding the effectivity of supplementary foods in managing children with MAM in low‐income, community‐based settings.

Furthermore, the barriers mothers experience when using prescribed supplementary foods for managing their children with MAM are unknown, as research conducted by others (Langlois, Suri, Walton, & Rogers, 2017; Nankumbi & Muliira, 2015), have not investigated this phenomenon, as health workers often assume that mothers will feed their malnourished children the supplementary foods they are issued with.

## 2 METHODS

### 2.1 Setting

The study was conducted in 12 parishes (serving as clusters) in the rural and peri‐urban districts of the Arua district in north‐western Uganda (Kajjura, Veldman, & Kassier, 2019a). The study community was conveniently selected because sorghum, maize and soy, the key ingredients of MSBP and CSB+ used in the cluster randomised control trial, are primary staples in the diet of residents in the district. An additional consideration was that records of the Arua Regional Referral Hospital reflected a high prevalence of MAM in the sampled parishes (Kajjura, Veldman, & Kassier, 2019b).

### 2.2 Study context

This study was embedded in a randomised control trial that compared the effectiveness of MSBP to CSB^+^ porridge in Arua district. The CSB+ was fortified with micronutrients whereas the MSBP was enriched with a biological enzyme active malt made from locally available sorghum. The MSBP, a predigested porridge as a result of the enzyme active malt could have enhanced the nutrient absorption of the already malnourished children digestive system, despite the fact that it was not fortified with micronutrients, making it comparable to CSB+ porridge, the current standard care for children with MAM in Uganda. The MSBP supplementary porridge is the first known of its kind to be formulated in Uganda, while making use of locally produced indigenous ingredients.

### 2.3 Intervention

Two hundred and four children aged 6 to 23 months diagnosed with MAM and residing in a rural community in Arua district, Uganda, were randomised to receive a malted sorghum‐based porridge (MSBP) in the treatment group (*n* = 104) and a fortified corn soy blend (CSB+) porridge as a supplementary food in the control group (*n* = 100; Kajjura et al., 2019a). The control supplementary porridge (manufactured by Reco Industries Limited, Kampala, Uganda) is used as the standard care for managing MAM in Uganda, whereas the treatment, developed for the purpose of the study, was manufactured from locally available malted and extruded ingredients that meet the nutritional needs of children with MAM (WHO, 2002). Both supplementary porridges had a similar protein and energy source in a ratio of 1:4 in order to increase nutrient density. Both supplementary porridge flours (MSBP and CSB+) had to be mixed with water and boiled for 15 min to render a viscosity suitable for infant and young child feeding.

Trained research assistants delivered the respective supplementary porridges to a mutually agreed upon community‐based site where mothers gathered on a weekly basis to receive nutrition education for the promotion of appropriate complementary feeding practices such as the importance of regular meal frequency and dietary diversity of household foods, as well as education on water and food safety. Mothers received the respective dry porridge rations once a week as a 1‐kg prepackaged bag free of charge. They also received a bar of soap once a month as a token of appreciation for their time as well as to improve their personal hygiene, thus preventing their children from contracting infectious disease such as diarrhoea. The trial hypothesised that there will be no significant difference in the anthropometric status (weight and length measures) of breastfed children aged 6 to 18 months diagnosed with MAM, irrespective of whether they were fed MSBP or CSB+ supplementary porridge for a period of 3 months.

This trial failed to reject the null hypothesis that supplementation with MSBP in conjunction with nutrition education of breastfeeding mothers will not result in a significant improvement in maternal feeding and hygiene practices, weight gain, weight‐for‐length z‐scores and haemoglobin levels compared with that of CSB+ in the management of children with MAM (Kajjura et al., 2019a & 2019b). This was underscored by the fact that the effect of MSBP on the nutritional status of children with MAM was not significantly different to that CSB+ at 5% confidence interval (Kajjura et al., 2019a).

The intervention shed light on maternal perceptions regarding the threat MAM posed to their children in relation to its severity and possible cues to action such as perceptions and barriers that could influence their decision to implement the intervention (French et al., 2012; Glanz, Rimer, & Viswanath, 2015). Maternal perceptions regarding the severity of their children's nutritional status served as a cue to action, as they were educated on the adverse health outcomes of untreated MAM among their offspring at baseline. A cue to action was therefore the perceived threat of untreated MAM. Other modifying factors implemented in the course of the 3‐month intervention included the weekly nutrition education session mothers received before the supplementary porridge rations were issued, as well as infant and young child weight gain. These modifying factors contributed to a sense of self‐efficacy to implement supplementation and improve complementary feeding practices (Glanz et al., 2015).

### 2.4 Study design

An exploratory study design with focus group discussions (FGDs) and in‐depth interviews (IDIs) (Marshall & Rossman, 2014; Maxwell, 2012) as qualitative research techniques were conducted. The study was conducted among mothers a week after completion of the 3‐month intervention to explore maternal perceptions of and barriers to feeding their children the respective supplementary porridges to minimise recall bias.

A detailed explanation of the study objectives, materials and methods was discussed with key members of the community where the study took place prior to finalisation of the research protocol to facilitate field mobilisation and obtain community input. The local government leadership in the Arua district provided permission to conduct the study within its parishes serving as clusters in the RCT. Prospective mother–child pairs meeting the inclusion criteria were individually informed about the purpose of the study in the local language.

### 2.5 Enrolment of mother–child pairs

**FIGURE 1:**
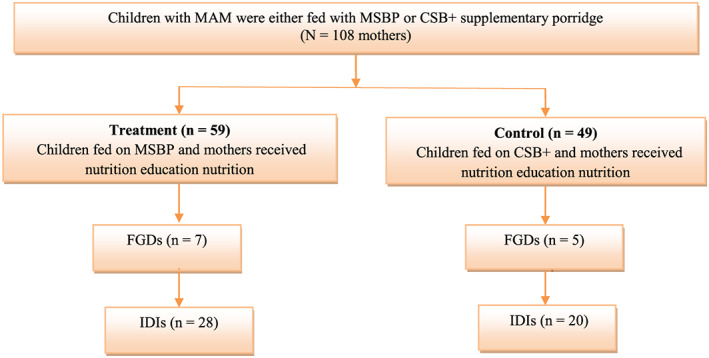
Enrolment of mother–child pairs. MSBP, malted sorghum base porridge; CSB+, fortified corn soy blend; IDIs, in‐depth interviews; FGDs, focus group discussions

Mother–child pairs that participated in the intervention were eligible for participation in this study. They were asked to assemble on a specific day at a central, mutually agreed upon site within the parish (cluster). The mothers in the selected trial clusters were approached and purposely sampled to participate in either an FGD or IDI forming part of study. For either FGDs or IDIs, an effort was made to recruit mothers that were diverse in terms of age, parity, marital status and level of education. The research assistants conducted the FGD first, following which IDI was conducted with another set of mothers. The order in which this was conducted was to prevent mothers participating in the FGDs to interact with those participating in the IDIs. Eligible participants were enrolled after signing an informed consent form. Twelve FGDs consisting of eight to nine mothers per group were subsequently recruited. Mothers with a hearing impairment and speech disorders were excluded due to potential communication difficulties. One hundred and eight mother–child pairs, of which 59 mother–child pairs were participating in the MSBP arm (treatment) of the trial, participated in seven FGDs, whereas 49 mothers who participated in the CSB+ arm (control) of the trial participated in five FGDs as illustrated in Figure 1. Mother–child pairs that were randomly allocated to either the treatment or control arm of the trial, were geographically separated by either several villages, or were parishes apart.

After completion of the FGDs, 48 mothers who did not participate in the FGDs were conveniently sampled to participate in the IDIs, with 28 that were enrolled in the MSBP (treatment) group and 20 that were enrolled in the CSB+ (control) groups respectively. Therefore, all mothers who met the study's inclusion criteria were given the opportunity to participate in a FGD or IDI. IDIs took place immediately after FGDs. During the recruitment of participants for the FGDs and IDIs, none of the potential participants refused to participate. The FGDs and IDIs were conducted until no additional concepts or themes emerged, indicative of data saturation (Saunders et al., 2018).

### 2.6 Research instruments

Construct validity of the research instruments were ensured by developing the FGD guides and structured IDI guides following extensive review of the relevant literature. Content validity was ensured by expert review of the FGD and IDI guides. In addition, input from an anthropologist was sought to review both guides. The two research instruments were developed in English and translated into the local language, Lugbarati. This was followed by back translation into English to ensure internal validity.

The FGD and IDI guides were piloted on mothers who participated in the supplementary feeding trial but were not purposively sampled for the qualitative study. The purpose of piloting was to determine whether the respective FGD and IDI guides were clear and not ambiguous, thereby ensuring reliability. Both discussion guides consisted of open ended questions to determine maternal perceptions of and barriers to feeding their children the respective supplementary porridges. The discussion topics included aspects such as asking participants to reflect on the 3‐month intervention period, to gauge perceptions and barriers pertaining to the children's weight gain, growth, feeding frequency, dietary diversity and satisfaction with the supplementary porridges that were provided. Participants were also asked to reflect on the reason(s) why they were willing to feed their children the respective supplementary porridges, as well as comment on interactions regarding the intervention they had with family members and members of the community during the intervention period.

### 2.7 Data collection

FGDs and IDIs were used to collect qualitative data regarding maternal perceptions of and barriers to feeding their children the respective supplementary porridges forming part of the management of MAM (Marshall & Rossman, 2014; Maxwell, 2012). The FGD members were asked to assemble on a specific day at a central, mutually agreed upon, site within the parish. FGDs were conducted by two trained research assistants serving as the facilitator and scribe in Lugbarati, the local language spoken by mothers. The research assistants were fluent in Lugbarati and resided in the Arua district where the mother–child pairs were recruited from. Each FGD had an approximate duration of 1 h. The scribe took notes regarding verbal and nonverbal cues exhibited by mothers in the course of discussions. IDIs were also conducted in Lugbarati. Individual FGDs were terminated once data saturation was reached (Saunders et al., 2018). At the conclusion of the FGDs and IDIs, the focus group facilitator and interviewer, respectively, moderated the scribed content of the notes taken by the scribe to determine assertion and meaning with mothers to enhance face validity. All FGDs and IDIs were tape recorded to facilitate verbatim transcription and translation was carried out by the research assistants. Participant validation was ensured, by concurring with focus group participants and mothers who participated in IDIs at the conclusion of discussions and interviews, respectively.

### 2.8 Data coding and analysis

FGD and IDI transcripts were transcribed verbatim from audio digital recordings in Lugbarati. Following transcriptions in Lugbarati, translation into English took place. The primary researcher, focus group facilitator, interviewer and scribe were independently involved in manually coding transcripts twice to generate a preliminary coding framework. This process helped to identify common themes and latent content relevant to the study objective. The English text was condensed to meaning units that were coded and classified into categories from which themes emerged. This approach enhanced the quality of the coding matrix and minimised intercoding variability, thus ensuring confirmability and reliability (Schreier, 2012). The codes guided content analysis to explore themes related to maternal perceptions, as well as barriers to intervention implementation that affected their ability and likelihood of taking action, despite knowledge gained as part of the intervention. Subsequently, six themes emerged as commonly held perceptions regarding the supplementary porridges, whereas 13 themes provided insight into the barriers to intervention implementation mothers experienced. Data were subsequently entered into Atlas.ti software version 5.2 to facilitate electronic coding according to identified themes using an inductive approach (Elo et al., 2014; Graneheim & Lundman, 2004) to explore maternal perceptions and barriers to feeding their children the respective supplementary porridges for the management of MAM. The main themes forming the core perceptions and barriers were then connected to one another to explain how mothers viewed the use of MSBP or CSB+ to treat children with MAM. In addition, relevant verbatim quotes were identified to support a theme that emerged during the process of content analysis.

### 2.9 Ethical considerations

This study was conducted in according to the guidelines laid down in the Helsinki and all procedures involving human subjects. Ethical approval was obtained from the Biomedical Research Ethics Committee (BE218/18) of the University of KwaZulu‐Natal, South Africa, Higher Degrees, Research and Ethics Committee (11353/394) of the School of Public Health, Makerere University, and Uganda National Council of Science and Technology (4705). All study participants signed an informed consent form in Lugbarati prior to participation. Where participants were illiterate, they made a thumb print on the consent form. All participants were guaranteed confidentiality and anonymity as they were assigned a coded number for identification purposes.

## 3 RESULTS

### 3.1 Participant characteristics

The results in Table 1 show that participants were between 18 and 50 years of age. Of the 108 mothers, 84.3% (*n* = 91) were younger than 35 years, 75.9% (*n* = 82) were traditionally married with the remaining 24.1% (*n* = 26) having a religious ceremony. The majority (85.2%; *n* = 92) had a primary school education or no formal education, while 63.0% (*n* = 68) had three or more children. The characteristics of participants who fed their children MSBP compared with those who fed CSB+ did not differ significantly.

**TABLE 1:** Participants' characteristics

Characteristics	MSBP group *n* (%)	CSB+ group *n* (%)	*P* value (z‐test)
Age of mother (years)			
<35	49 (83.0)	42 (85.7)	0.231
≥35	10 (17.0)	7 (14.3)	
Mother's parity (# children)			
<3	23 (39.0)	17 (34.7)	0.647
≥3	36 (61.0)	32 (65.3)	
Mother's education level			
≤Primary education	48 (64.4)	44 (75.5)	0.261
≥Secondary education	11 (18.6)	5 (10.2)	
Mother marital status			
Married	44 (74.6)	38 (77.6)	0.623
Single	15 (24.0)	11 (22.4)	

### 3.2 Maternal perceptions of using supplementary porridges

The health indicators perceived by mothers are summarised in Table 2. Mothers reported that they were satisfied with the use of MSBP or CSB+ to feed their children.

**TABLE 2:** Commonly mentioned healthy benefits

Health indicators	Mothers of MSBP group (*n* = 28)	Mothers of CSB+ group (*n* = 20)
Children gained weight	27	18
Children's appetite improved	22	16
Children engaged in active play	25	17
Children were rarely ill	18	13
Children's skin looked healthy	16	11
Children became healthy	26	17

Abbreviations: MSBP, malted sorghum base porridge; CSB+, fortified corn soy blend.

**TABLE 3:** Perceived barriers to using supplementary porridges

*Barriers to using supplementary porridges*
Lack of social support resulted in demanding household chores Shortage of household food resulted in maternal hunger Inadequate level of knowledge regarding appropriate childhood feeding practices due to low level of education Inadequate household income influenced quality of feeding Limited livelihoods and unemployment
*Perceptions of using supplementary porridges*
Supplementary porridge improved engagement in active play Supplementary feeding complemented breastfeeding Weight gain of children motivated intervention completion Supplementary porridge satisfied children's hunger Illness occurrence among children reduced Maternal stress related to inadequate food for children reduced Mothers' household chores increased by the preparation of supplementary porridge

Abbreviations: IDI, in‐depth interview; FGDs, focus group discussions.

### 3.3 Improved appetite

An improvement in children's appetite in the course of the intervention was mentioned as an advantage of feeding their children the respective supplementary porridges. These findings are illustrated by the following quotes:

This child was without strength, … the appetite for food was not there, but now because of taking this porridge, it has brought him appetite for food. He now eats all foods than before, makes his weight to increase and now my child is well. (FGD mother, CSB+)

My child before the porridge, appetite for household foods in addition to breast milk was low…. after taking the porridge, he has a lot of appetite for breast milk and he feeds happily with good appetite till he is satisfied … and his weight has increased. (FGD mother, MSBP)

The results show that irrespective of which supplementary porridge (either MSBP or CSB+) was fed to participating children, an improvement in appetite and weight gain was mentioned.

### 3.4 Perceived health benefits

The perceived health benefits mentioned by mothers are summarised in Table 2. An improvement in children participating in active play, satisfying hunger and an increased feeding frequency as a result of nutrition education, as well as the contribution to dietary diversity made by the supplementary porridges, emerged as advantages of the intervention. Mothers also reported that supplementary porridges were a source of energy for their children, and that it contributed to healthy looking skin and growth.

The health of my child was not good, he was in and out of hospital, his body was pale, he had brown hair but now he is healthy with good appetite, participating in active play and less sickly after taking the porridge. (IDI mother, CSB+)

My child was weak, with rough skin, and brown hair, but now she stands on her own, and her hair is dark with a smooth face”. She used to be sickly but he does not fall ill often and participates in active play. (IDI mother, MSBP)

Mothers reported that feeding children the supplementary porridge resulted in growth, as was evident from the weight gained (Table 2). Furthermore, participant motivation to feed their children the supplementary porridge was mainly attributed to the fact that they were weak and underweight before the intervention commenced. However, once their children started participating in the intervention, they became healthy and gained weight in due course. This is illustrated by the following quote:

I did not expect my child to improve like this; He is now healthy, gained weight and is more active. (IDI mother, MSBP)

In addition, it was perceived that underweight prior to participation in the intervention, resulted in a lack of energy, and the necessary strength required for active play (Table 2). Mothers reported that the supplementary porridge resulted in weight gain, engagement in active play, reduced illness, improved skin health, and contributed to overall health of their children. Two of the mothers participating in the IDI mentioned that

Frequent illness and less strength for active play are suggestive that the child does not have enough food intakes in the body. (IDI mother, CSB+)

Child health was not good before the porridge … the child that was once weak is now healthy and engagement in active play. (IDI mother, MSBP)

Collectively, all the mothers that were recruited for IDIs mentioned a decrease in the frequency of infectious disease such as diarrhoea among children by the time the intervention reached its conclusion.

### 3.5 Reduced demand for breastfeeding

Mothers participating in IDIs of both supplementary groups reported an improvement in their children's feeding regime compared to what it was prior to the intervention. Due to supplementary feeding, in conjunction with continued breastfeeding, feeding became more structured and children were seemingly not hungry as often. Therefore they demanded breastfeeding less frequently. During IDIs, mothers seemed satisfied with breastfeeding and supplementary feeding regimes as a result of the intervention. Some of the themes are illustrated by the following quote:

The porridge has made my child look good, I'm so happy for his weight to increase and also he does not breastfeed so often like he used to before the porridge intervention. Often, when I don't give her the porridge, she over breastfeeds me, and I don't get any break to rest. (IDI mother, MSBP)

### 3.6 Maternal barriers to using supplementary porridges

Mothers participating in FGDs from both the treatment (MSBP) and control (CSB+) groups explained that barriers to the implementation of the supplementary porridge intervention included household chores such as subsistence farming, gathering food, as well as food preparation for which they received minimal support from household members. These chores that take up a lot of the mother's time of being a barrier to supplementary feeding as is illustrated by the following quote:

As a mother you start working in the morning with gardening, gathering food, washing clothes, bathing children, going to the market, and cooking food, which takes a lot my time before I feed my child. (IDI mother, MSBP)

In addition, a lack of time for feeding children due to household responsibilities was commonly mentioned as a reason for partial implementation of the intervention protocol, such as feeding children twice instead of three times a day. The barrier theme is illustrated by the following quote:

My family is large, with a lot of house work that needs me, such as gardening and preparing food, which takes a lot of my time before I feed my child the porridge. (FGD mother, MSBP)

During IDIs, irrespective of whether mothers were randomised into the treatment or control arm of the trial, they reported that during the intervention period, they received minimal household support in the management of their children with MAM. Maternal stress emerged as a barrier to caring for their children, stemming from having to do household chores, a lack of time and money, food insecurity, and a lack of adequate knowledge regarding appropriate childhood feeding practices due to a lack of formal education. Conversely, the nutrition education of mothers as a component of the intervention improved the dietary diversity of their children as is illustrated by the following quote.

Before this intervention, buying food was so difficult for me to feed my children adequately. Because I don't work to get money, but after learning of cheaper foods like silver fish, oranges, fruits it became easy for me to feed my children on a variety of foods. (FGD mother, CSB+)

### 3.7 Altered perceptions of community members

Mothers participating in FGDs of both the control and treatment groups reported that members of the community had mixed feelings about the intervention. Some community members remarked that supplementary foods are intended for households that failed to take care of the nutritional needs of their children. However, this comment did not deter mothers from continuing with the intervention. For example, a month after the intervention was implemented, the same members of the community with healthy children enquired from participating mothers how to go about joining the intervention. The latter phenomenon encouraged mothers in both supplementary groups to continue with the intervention, in addition to the tangible evidence they observed such as an improved appetite, weight gain and participation in active play of their children. This is reflected by the following quotes:

My neighbour was happy because my child's body was wrinkled before but now it is smoot…his hair has changed from brown to black … .the father and my mother are both happy and do not want me to stop giving porridge because my child's health is good’...my child is engaged in active play. (FGD mother, MSBP)

When the porridge ration began, people in my community despised me saying that porridge was for those with no food at home but later, the same people saw positive changes such as weight gain and participation in active play in my child, and wanted to pick the porridge though it was too late. (FGD mother, CBS+)

Collectively, mothers who participated in FGD and IDI reported that the outcome of the intervention was positive, as it resulted in growth as was evident from the weight their children gained. Hence, the negative feedback they received from members of the community did not serve as a deterrent to participation. In addition, participants from both groups reported that their motivation for feeding their children, the respective supplementary porridges, was predominantly related to the fact that before the intervention commenced, their children were weak and underweight but became healthy and gained weight following enrolment.

## 4 DISCUSSION

The aim of this qualitative study was to determine maternal perceptions of, and barriers to feeding their children the respective supplementary porridges that formed part of a 3‐month intervention. Data were collected by means of FGD, and IDI a week after the intervention was concluded.

### 4.1 Maternal perceptions regarding use of supplementary porridges

In the current study, maternal perceptions that the supplementary porridges contributed to weight gain, were held by mothers, irrespective of whether they were randomised into the treatment (MSBP) or control (CSB+) arms of the trial. The positive perceptions held by mothers were indicative of a belief in the use of both MSBP and CSB+ as supplementary porridges in the management of their children with MAM, as mothers from both groups perceived that their children gained weight following enrolment in the intervention and that weight gain contributed to good health. It therefore suggests that participating mothers were confident that the supplementary porridges would contribute to children's growth as was observed from their weight gain. This finding compares favourably to that reported by a systematic review concluding that nutrient dense foods such as ready‐to‐use therapies are needed by children with MAM to facilitate growth (Briend et al., 2015). In addition, this finding is also consistent with studies conducted in Malawi and South Africa, respectively, where rural mothers held a similar perception regarding the use of supplementary food (Flax et al., 2009; Nor et al., 2012). The results are also consistent with studies (Iuel‐Brockdorf et al., 2015; Langlois et al., 2017) that made use of malted and fortified cereal‐legume based supplementary foods fed to children with MAM where children's nutritional needs for healthy growth was met (Briend et al., 2015; Dewey & Adu‐Afarwuah, 2008). Overall, both the MSBP and CSB+ supplementary porridges were perceived as beneficial to participating children.

### 4.2 Improved appetite

An improvement in appetite observed among children was perceived as positive by both mothers from the MSBP and CSB+ groups. In addition, it was also perceived to be an indicator of improved health (Lassi, Das, Zahid, Imdad, & Bhutta, 2013) which mothers valued. The outcome related to an improvement in appetite, is echoed by studies conducted in Burkina Faso and Uganda, where mothers/caretakers experienced an improvement in children's appetite as a result of appropriate supplementary feeding practices with corn soya blends (Ickes et al., 2012; Iuel‐Brockdorf et al., 2015).

### 4.3 Health benefits

Indicators of children's health, namely, weight gain, an improved appetite, participation in active play, reduced prevalence of illness and an improvement in skin health, were not only observed among participating children with MAM but also became evident when mothers compared their children to those in the community who were not diagnosed with MAM. It would therefore seem that provision of supplementary foods in conjunction with nutrition education markedly improved the health indicators of children with MAM. In addition, it would seem that nutrition education creates an awareness of mothers' experiences while caring for their malnourished children receiving supplementary foods (Ashworth & Ferguson, 2009; Ickes et al., 2015). In this study, nutrition education could have improved mothers' ability to care for their moderately malnourished children (Kajjura, Veldman, & Kassier, 2019c).

An improvement in children's participation in active play was often mentioned by FGD participants, irrespective of whether their children were supplemented with MSBP or CSB+. This observed improvement in their children's health could have resulted in an alleviation of apathy that in turn contributed to an increase in active play. Mothers also reported that their children showed an improvement in skin health. This perception was also held by mothers, irrespective of which supplementary porridge their children received. This finding is indicative of the finding that a child's skin health is related to an increase in food intake (Aguilar, Wengreen, Lefevre, Madden, & Gast, 2014). Thus, an improvement in children's health was evident from their healthy looking skin.

Furthermore, a reduction in the prevalence of illness after the intervention reached its conclusion, irrespective of the supplementary porridge received, was reported by mothers. This implies that an adequate consumption of the respective supplementary porridges enhanced children's immunity. The latter is similar to the findings of a study conducted in India, where it was shown that an improvement in nutritional status was associated with infrequent childhood infections (Sood & Sood, 2015).

An improvement in the indicators of childhood health that were observed in this intervention could have motivated mothers to continue with the intervention. A study conducted in western Uganda also found that an improvement in growth influences caregiver willingness to feed a supplement (Ickes et al., 2012). Therefore, interventions with the aim of improving child health indicators in Uganda may benefit from including supplementary porridges (Ickes et al., 2015).

### 4.4 Maternal barriers to using supplementary porridges

Mothers mentioned that household chores competed with their ability to comply with the intervention protocol, especially when it came to the frequency of feeding their children the respective supplementary porridges, as chores resulted in a lack of time to feed children regularly. Another barrier reported by mothers was related to a lack of social support from household members. Although barriers such as a lack of time to feed children, resulting in an inability to meet their nutritional needs have been reported in both Uganda (Ickes et al., 2012; Ickes et al., 2018; Nankumbi & Muliira, 2015) and Bolivia (Tadesse et al., 2015), the use of extruded and malted flours as was the case in the current intervention, reduced cooking time. This could have contributed to mothers completing the intervention, in addition to health benefits their children reaped.

Conversely, this study reported a lack of adequate knowledge regarding appropriate childhood feeding practices due to a lack of formal maternal education, as well as a lack of money to procure food due to their low purchasing power. It is therefore important to take cognisance of factors such as inadequate household income, as it has an impact on household food insecurity, available time for child care, as well as a lack of social support from family members and the community at large. These barriers have the potential to significantly impair the ability of mothers with malnourished children from complying with supplementation protocols (Ickes et al., 2018).

### 4.5 Perceptions of community regarding the use of supplementary foods

The perception held by members of the community that supplementary foods are meant for households that failed to take care of their children was indicative of certain members of the community stigmatising mothers of children with MAM based on their participation in the intervention. In addition, this opinion held by members of the community points towards a lack of knowledge and insight regarding the importance of supplementary foods in the management of children with MAM. This finding is supported by the fact that a lack of knowledge not only affects childhood feeding practices (Ickes et al., 2015; Jones et al., 2012; Nankumbi & Muliira, 2015) but could possibly have an effect on perceptions held towards childhood feeding.

## 5 CONCLUSION

This study investigated the perceptions of and barriers to the use of supplementary porridges for a period of 3 months, in conjunction with nutrition education of mothers of moderately malnourished children. Furthermore, it documented that the decision of mothers to feed their children the supplementary porridges they were issued with, and comply with the intervention, was based on whether they believed in the ability of these supplementary foods to improve the nutritional status of their children suffering from MAM. However, the mothers that were purposively sampled for participation in this qualitative study may not have been representative of the views of all mothers that participated in the intervention. Participating mothers held the perception that MSBP and CSB+ supplementation of their children with MAM resulted in weight gain as an indicator of growth, improved appetite, and resulted in participation in active play, an improvement in skin health and a reduced prevalence of illness. As these perceptions were held by mothers, irrespective of the supplementary porridge their children received, it is possible that participation in a supplementary feeding intervention and the resultant health outcomes of moderately malnourished children receiving the supplementary food is a motivating factor for continued participation. Therefore, the health benefits that can be attributed to the use of supplementary foods in the management of MAM should be emphasised by health workers during home visits and information sessions involving the community at communal gathering places.

Perceived barriers that impaired mothers' ability to comply with the supplementation protocol included household chores, a lack of time to regularly feed their children, limited household income and household food insecurity. Other barriers included a lack of social support from household members and stigmatisation by members of the community. Perceived maternal barriers to using supplementary foods in the management of children with MAM need to be addressed by intervention planners and policy makers, as it could contribute to optimising the health outcomes of children participating in interventions of this nature.

Hence, results generated by this qualitative study provides insight into mothers' perceived benefits gained from participating in a supplementary feeding intervention targeting their breastfed children with MAM receiving a supplementary porridge, as well as barriers to intervention implementation and successful outcomes. Thus, knowledge regarding these enablers and barriers to implementation could benefit health care professionals and programme managers in the planning stage of a supplementary feeding intervention targeting children diagnosed with MAM to enhance intervention efficacy. Health workers responsible for issuing supplementary food rations should make a concerted effort to educate mothers regarding the potential barriers they may face when providing optimal care to their malnourished children. In addition, mothers should also be made aware that they could face stigmatisation and a lack of support from household members as well as the community to prevent these potential barriers from interfering with intervention compliance.

## CONFLICTS OF INTEREST

Authors declare that they do not have any financial or nonfinancial competing interests for this paper.

## CONTRIBUTIONS

Richard Bazibu Kajjura designed the research, performed data collection, data management, data analysis, and result interpretation, and drafted the manuscript. Frederick Johannes Veldman and Susanna Maria Kassier revised the study designed, interpreted the results and revised the draft manuscript. All authors read and approved the final manuscript.
